# Prediction algorithm for gastric cancer in a general population: A validation study

**DOI:** 10.1002/cam4.6629

**Published:** 2023-10-19

**Authors:** Martin C. S. Wong, Eman Yee‐man Leung, Sarah T. Y. Yau, Sze Chai Chan, Shaohua Xie, Wanghong Xu, Junjie Huang

**Affiliations:** ^1^ The Jockey Club School of Public Health and Primary Care, Faculty of Medicine Chinese University of Hong Kong Hong Kong SAR China; ^2^ Centre for Health Education and Health Promotion, Faculty of Medicine Chinese University of Hong Kong Hong Kong SAR China; ^3^ School of Public Health The Chinese Academy of Medical Sciences and Peking Union Medical College Beijing China; ^4^ School of Public Health The Peking University Beijing China; ^5^ School of Public Health Fudan University Shanghai China; ^6^ Department of Molecular medicine and Surgery Karolinska Institutet Sweden

**Keywords:** aspirin, gastric cancer, predictors, proton pump inhibitors, risk score

## Abstract

**Background:**

Worldwide, gastric cancer is a leading cause of cancer incidence and mortality. This study aims to devise and validate a scoring system based on readily available clinical data to predict the risk of gastric cancer in a large Chinese population.

**Methods:**

We included a total of 6,209,697 subjects aged between 18 and 70 years who have received upper digestive endoscopy in Hong Kong from 1997 to 2018. A binary logistic regression model was constructed to examine the predictors of gastric cancer in a derivation cohort (*n* = 4,347,224), followed by model evaluation in a validation cohort (*n* = 1,862,473). The algorithm's discriminatory ability was evaluated as the area under the curve (AUC) of the mathematically constructed receiver operating characteristic (ROC) curve.

**Results:**

Age, male gender, history of *Helicobacter pylori* infection, use of proton pump inhibitors, non‐use of aspirin, non‐steroidal anti‐inflammatory drugs (NSAIDs), and statins were significantly associated with gastric cancer. A scoring of ≤8 was designated as “average risk (AR)”. Scores at 9 or above were assigned as “high risk (HR)”. The prevalence of gastric cancer was 1.81% and 0.096%, respectively, for the HR and LR groups. The AUC for the risk score in the validation cohort was 0.834, implying an excellent fit of the model.

**Conclusions:**

This study has validated a simple, accurate, and easy‐to‐use scoring algorithm which has a high discriminatory capability to predict gastric cancer. The score could be adopted to risk stratify subjects suspected as having gastric cancer, thus allowing prioritized upper digestive tract investigation.

## 1 INTRODUCTION

Gastric cancer is a significant global health issue, accounting for approximately 5.6% of all new cancer cases globally in 2020.^1^ It is associated with high mortality rates, contributing to 7.7% of cancer‐related deaths worldwide. The prognosis for gastric cancer is particularly poor, with a 5‐year survival rate of dropping from 70% for localized cases to 6% for cases diagnosed at a distant stage.^2^ Given the alarming statistics, it is imperative to develop an effective scoring system that can accurately predict the risk of gastric cancer to facilitate early detection, prompt intervention, and reduce mortality.

Previous studies have identified several demographic factors associated with gastric cancer and utilized them in risk prediction models.^3^ For example, it has been observed that male individuals had a higher incidence than females, with this difference being more pronounced in the elderly population.^4^ Additionally, the use or non‐use of certain chronic medications has been linked to an increased risk of gastric cancer. It was found that the use of proton pump inhibitors would increase the chance of having gastric cancer^5^; while the use of non‐steroidal anti‐inflammatory drugs (NSAIDS),^6^ aspirin,^7^ and statin^8^ has been found to significantly reduce the risk of gastric cancer incidence.

Previous studies have reviewed and evaluated the efficiency of available prediction models. However, they have a high risk of bias due to methodologic limitations, and their generalizability to other settings remains uncertain.^3^ Also, some models were developed based on a relatively small sample size.^9^ The present study aims to address these gaps by devising and validating a simple and accurate scoring algorithm that is capable of discriminating and predicting gastric cancer based on a large population dataset. We anticipate that such an algorithm will enable risk stratification of individuals suspected of having gastric cancer and facilitate the prioritization of upper digestive tract investigation. Additionally, the external validity of the scoring algorithm can be examined in diverse population groups through further studies.

## 2 MATERIALS AND METHODS

### 2.1 Ethics statement

This study was approved by the Survey and Behavioural Research Ethics Committee of the Chinese University of Hong Kong (No. SBRE‐20‐882). Inform consent was waived by the Committee as this was a retrospective analysis of anonymized data.

### 2.2 Study setting

In this study, data were extracted from Hospital Authority Data Collaboration Lab (HADCL), which is a platform providing access to an electronic healthcare database that consists of patient demographic data, clinical diagnoses, procedures, drug prescriptions, and laboratory results from all public hospitals and clinics in Hong Kong. It represents in‐patient and out‐patient data of about 80% of the 7.49 million people in our locality. We have previously validated the database and reported a high level of completeness of patients' demographic profiles (100%) and prescription details (99.8%).^10^ The data on comorbidities were coded by International Classification of Diseases Ninth Revision, Clinical Modification (ICD‐9‐CM) in CDARS, which have been validated to be 99% accurate with regard to clinical, laboratory, imaging, and endoscopy results from the electronic medical records.^11^, ^12^, ^13^ Sociodemographic data including the year of birth; sex; previous history of *Helicobacter pylori* infection; use of proton pump inhibitors, histamine receptor‐2 antagonists (H_2_ receptor blockers), aspirin, NSAIDs, and statins; and histopathology findings of suspected gastric lesions were collected. Coexisting medical conditions in each patient were also extracted with the use of all relevant ICD‐9‐CM diagnosis and procedure codes. The present study was performed in accordance with the ethical guidelines of the Declaration of Helsinki. The study was approved by the Survey and Behavioral Research Ethics Committee of the Chinese University of Hong Kong.

### 2.3 Study subjects

We included all adults aged between 18 and 70 years who have received oesophago‐gastroduodenoscopy (OGD) in the Hospital Authority of Hong Kong from 1997 to 2018, as documented in the database. For individuals who received more than one OGD in the study period, we used findings from the earliest OGD to avoid over‐representation of a certain group of subjects. We identified patients with gastric cancer using the following criteria: (1). having an ICD‐9 coding of 157.0, 157.1, 157.2, 157.3, 157.4, 157.8 or 157.9; 2). gastric tissue biopsy results showing “adenocarcinoma,” “carcinoma,” or “lymphoma”; 3). OGD results reporting “carcinoma” or “lymphoma”. Individuals who did not meet these criteria were considered as control subjects.

### 2.4 Oesophago‐gastroduodenoscopy (OGD)

OGD was performed by both surgeons and physicians, and conscious sedation was provided by the endoscopists. The procedures were performed either by specialists or by trainees with at least 4 years of experience in OGD under supervision. All patients were given eight puffs of 10% topical xylocaine before the procedure. The decision for conscious sedation was up to the endoscopists' discretion, depending on the patients' condition, the anticipated difficulty of the procedure, and the expected duration of the investigation. Conscious sedation was provided by either intravenous midazolam with or without intravenous pethidine. The endoscopy team included the chief endoscopist, endoscopy nurse and airway nurse.

### 2.5 Derivation and validation cohorts

We randomly split this cohort into a derivation (*n* = 4,347,224) and validation cohort (*n* = 1,862,473) (Figure S1) in a 7:3 ratio. The proposed study included consecutively recruited patients. We assumed 25% as the point prevalence of individual risk factors and 0.1% as the prevalence of gastric cancer in the derivation set, as in the Asia–Pacific Colorectal Screening (APCS) study performed by Yeoh et al.^14^ Based on these assumptions, a sample size of more than 6.2 million could attain a power of >99% and detect a risk factor with an odds ratio of 2.0 at a significance level of *p* < 0.05, according to “Sample size and optimal design for logistic regression with binary interaction” (2008) published by Demidenko in *Statistics in Medicine* (27:36–46). We examined the association between detection of gastric cancer and each predictor in the derivation cohort using Pearson's chi‐square test. We included parameters based on sociodemographic parameters, past medical conditions, and use of medications in the risk score.

### 2.6 Development of the risk scores

A multivariable regression analysis of all variables that predict gastric cancer with statistical significance (*p* < 0.05) in the univariable analysis was performed. The outcome of the multivariable analysis was the detection of gastric cancer through OGD. Meanwhile, the adjusted odds ratios (AORs) were calculated using all significant variables according to a binary logistic regression model of the derivation cohort. The same set of risk factors for gastric cancer were also modelled separately among those who were referred to OGD from outpatient consultations or inpatient hospitalizations. A multilevel model was also performed to assess the effect of potential strata by clustering effects and year of OGD in addition to the multiple logistic regression model.

The scoring system was based on the regression coefficients of a logistic model.^15^ Each individual subject's score was the sum of the scores of the identified predictor variables, on which basis we formulated a scoring algorithm that takes each weighted independent variable as an input. The discriminatory ability of the algorithm was evaluated as the area under the curve (AUC) of the mathematically constructed receiver operating characteristic (ROC) curve. The actual predictive ability of the risk score was computed as the concordance statistic (c‐statistic). We compared the risk‐scoring system critically with internationally published models.

### 2.7 Statistical analysis

For each score in the derivation cohort, the predicted proportion of gastric cancer was calculated. Scores with magnitudes equal to or less than the average proportion of gastric cancer were classified as “average risk” (AR), whereas those with magnitudes statistically exceeding the average proportion were assigned to the “high risk” (HR) category. The Cochran–Armitage trend test was performed to determine whether an increase in the gastric cancer proportion as a function of the risk score was statistically significant. We used the Hosmer–Lemeshow goodness‐of‐fit statistics to assess the reliability of the final prediction algorithm. As a statistical criterion, *p* > 0.05 indicated an acceptably strong relationship between the predicted and observed risks. The number needed to screen (NNS), defined as the inverse of the outcome probability predicted by the regression model, was evaluated as a measure of the prospective resource requirements if the scoring system were applied clinically to refer HR participants to OGD for further work‐up. All statistical effects with a two‐sided *p* < 0.05 were deemed significant. All the statistical analyses conducted in this study were performed using R Statistical Software (version 3.5.2).^16^

## 3 RESULTS

### 3.1 Participant characteristics

The patient characteristics are similar in the derivation cohort and the validation cohort from OGDs conducted in 1997–2018 (Table 1). The average age of the study participants was 44.5 years with a male sex ratio of 44.7%. Only 0.01% suffered from previous history of *H*. *pylori* infection, possibly due to low testing rate; while cerebrovascular disease (1.5%) and ischemic heart disease (1.4%) were the most common comorbidities. The proportion of individuals who were using H_2_ blockers, NSAIDs, PPIs, statins, and aspirin was 3.0%, 2.9%, 1.8%, 1.1%, and 0.7%, respectively.

**TABLE 1:** Characteristics of patients in the derivation and validation cohorts.

Characteristics	Derivation cohort (*n* = 4,347,224)	Validation cohort (*n* = 1,862,473)	*p*‐value
Age in years, mean ± SD	44.52 ± 14.49	44.5 ± 14.48	0.102
Male sex, *n* (%)	1,944,860 (44.7)	832,345 (44.7)	0.274
*Helicobacter pylori* infection, *n* (%)	306 (0.01)	146 (0.01)	0.284
Ischemic heart disease, *n* (%)	60,304 (1.4)	25,546 (1.4)	0.128
Cerebrovascular disease, *n* (%)	64,986 (1.5)	27,750 (1.5)	0.643
Heart failure, *n* (%)	19,530 (0.4)	8416 (0.5)	0.655
Use of NSAIDs, *n* (%)	124,328 (2.9)	53,582 (2.9)	0.245
Use of aspirin, *n* (%)	29,323 (0.7)	12,571 (0.7)	0.951
Use of statins, *n* (%)	47,842 (1.1)	20,677 (1.1)	0.290
Use of H_2_‐blockers, *n* (%)	129,007 (3.0)	55,620 (3.0)	0.207
Use of PPIs, *n* (%)	77,468 (1.8)	33,261 (1.8)	0.740
Gastric cancer, *n* (%)	4402 (0.10)	1899 (0.10)	0.801

Abbreviations: H_2_‐blockers, histamine H_2_‐receptor antagonists; NSAIDs, non‐steroidal anti‐inflammatory drugs; PPIs, proton pump inhibitors; SD, standard deviation.

### 3.2 Independent predictors of gastric cancer in the derivation cohort

The proportion of gastric cancer (Table 2 and 3) was higher in male than female subjects (0.13% vs. 0.08%; crude odds ratio [cOR]: 1.69, 95% CI. 1.60–1.80); older people (0.01% in 18–39 years gradually increasing to 0.43%, cOR: 6.27 to 82.75), patients with previous history of *H. pylori* infection (2.61% vs. 0.10%, cOR: 26.53, 95% CI 13.14–53.58), and those with different chronic diseases (ischemic heart disease 0.29% vs. 0.10%, cOR: 2.98, 95% CI 2.57–3.47); cerebrovascular disease (0.30% vs. 0.10%, cOR: 3.03, 95% CI 2.62–3.50); and heart failure (0.24% vs. 0.10%, cOR 2.34, 95% CI 1.75–3.13). Study participants who were using chronic medications were significantly more likely to have gastric cancer diagnosed (NSAIDs 0.41% vs. 0.09%, cOR: 4.45, 95% CI 4.08–4.88; aspirin 0.38% vs. 0.10%, cOR: 3.86, 95% CI 3.19–4.65; statins 0.32 vs. 0.10%, cOR: 3.27, 95% CI 2.78–3.84; H_2_ blockers 0.49% vs. 0.09%, cOR: 5.50, 95% CI 5.06–5.99; PPIs 0.90% vs. 0.09%, cOR: 10.51, 95% CI 9.69–11.40; all *p* < 0.001).

**TABLE 2:** Prevalence of gastric cancer in the derivation cohort by risk factors.

	All subjects	Gastric cancer (*n* = 4402)	
Risk factors	Prevalence (%)	Prevalence (%)	*p*‐value
Sex			<0.001
Female	2,402,364 (55.3)	1857 (0.08)	
Male	1,944,860 (44.7)	2545 (0.13)	
Age, years			<0.001
18–39	1,686,386 (38.8)	89 (0.01)	
40–49	924,464 (21.3)	306 (0.03)	
50–55	536,387 (12.3)	491 (0.09)	
56–60	461,589 (10.6)	874 (0.19)	
61–65	404,940 (9.3)	1192 (0.29)	
66–70	333,458 (7.7)	1450 (0.43)	
*Helicobacter pylori* infection			<0.001
No	4,346,918 (99.99)	4394 (0.10)	
Yes	306 (0.01)	8 (2.61)	
Ischemic heart disease			<0.001
No	4,286,920 (98.6)	4225 (0.10)	
Yes	60,304 (1.4)	177 (0.29)	
Cerebrovascular disease			<0.001
No	4,282,238 (98.5)	4209 (0.10)	
Yes	64,986 (1.5)	193 (0.30)	
Heart failure			<0.001
No	4,327,694 (99.6)	4356 (0.10)	
Yes	19,530 (0.4)	46 (0.24)	
Use of NSAIDs			<0.001
No	4,222,896 (97.1)	3894 (0.09)	
Yes	124,328 (2.9)	508 (0.41)	
Use of aspirin			<0.001
No	4,317,901 (99.3)	4290 (0.10)	
Yes	29,323 (0.7)	112 (0.38)	
Use of statins			<0.001
No	4,299,382 (98.9)	4248 (0.10)	
Yes	47,842 (1.1)	154 (0.32)	
Use of H_2_‐blockers			<0.001
No	4,218,217 (97.0)	3770 (0.09)	
Yes	129,007 (3.0)	632 (0.49)	
Use of PPIs			<0.001
No	4,269,756 (98.2)	3702 (0.09)	
Yes	77,468 (1.8)	700 (0.90)	

Abbreviations: H_2_‐blockers, histamine H_2_‐receptor antagonists; NSAIDs, non‐steroidal anti‐inflammatory drugs; PPIs, proton pump inhibitors.

**TABLE 3:** Univariate and multivariable predictors of gastric cancer in the derivation cohort.

		N0	N1	N2
Risk factors	Crude OR (95% CI)	Adjusted OR (95% CI)	Adjusted OR (95% CI)	Adjusted OR (95% CI)
Age, years				
18–39	1	1	1	1
40–49	6.27 (4.96–7.94)	6.35 (5.02–8.05)	6.36 (5.02–8.05)	6.36 (5.07–7.97)
50–55	17.36 (13.85–21.76)	16.54 (13.19–20.73)	16.57 (13.22–20.77)	16.55 (13.21–20.75)
56–60	35.95 (28.90–44.71)	32.41 (26.05–40.33)	32.50 (26.12–40.43)	32.45 (26.08–40.37)
61–65	55.94 (45.10–69.39)	49.21 (39.65–61.08)	49.43 (39.83–61.34)	49.30 (39.73–61.18)
66–70	82.75 (66.80–102.50)	70.76 (57.06–87.74)	71.26 (57.49–88.35)	70.96 (57.24–87.96)
Male sex	1.69 (1.60–1.80)	1.49 (1.40–1.59)	1.50 (1.41–1.59)	1.50 (1.41–1.59)
*Helicobacter pylori* infection	26.53 (13.14–53.58)	2.15 (1.06–4.38)	2.13 (1.05–4.34)	2.10 (1.03–4.29)
Ischemic heart disease	2.98 (2.57–3.47)	1.03 (0.87–1.20)	‐	‐
Cerebrovascular disease	3.03 (2.62–3.50)	1.14 (0.99–1.33)	‐	‐
Heart failure	2.34 (1.75–3.13)	0.74 (0.55–1.00)	0.76 (0.57–1.02)	‐
Use of NSAIDs	4.45 (4.05–4.88)	0.52 (0.45–0.61)	0.49 (0.43–0.57)	0.49 (0.43–0.57)
Use of aspirin	3.86 (3.19–4.65)	0.58 (0.46–0.73)	0.59 (0.47–0.74)	0.58 (0.46–0.72)
Use of statins	3.27 (2.78–3.84)	0.45 (0.37–0.55)	0.45 (0.37–0.55)	0.45 (0.37–0.54)
Use of H_2_‐blockers	5.50 (5.06–5.99)	0.86 (0.71–1.03)	‐	‐
Use of PPIs	10.51 (9.69–11.40)	10.27 (8.74–12.06)	9.40 (8.29–10.65)	9.38 (8.28–10.63)
Area under the curve (AUC)		0.8329	0.8334	0.8335

Abbreviations: CI, confidence interval; H_2_‐blockers, histamine H_2_‐receptor antagonists; NSAIDs, non‐steroidal anti‐inflammatory drugs; OR, odds ratio; PPIs, proton pump inhibitors.

### 3.3 Multivariate regression models

We constructed three models with N_0_ entering all covariates; N_1_ including only those variables found to be significant in univariate analysis; and N_3_ using significant variables in binary logistic regression analysis. The final model (N_3_) consisted of age (aOR: 6.36–70.96), male gender (aOR: 1.50, 95% CI 1.41–1.59), *H. pylori* infection (2.10 (1.03–4.29), and use of four chronic medications (aspirin, NSAIDs, and statins: aOR ranged from 0.45 to 0.58; PPI: 9.38, 95% CI 8.28–10.63). The c‐statistics (0.834, 95% CI 0.822–0.845) was the highest when N_3_ was used as a risk stratification system.

### 3.4 Development of the risk score

According to the AORs from the derivation cohort, the following variables were used to assign scores to each screening participant (Table 4): age 18–39 years (0), 40–49 years (2), 50–60 years (3), 61–70 years (4); male gender (1), female gender (0); previous history of *H. pylori* infection (1); not using NSAIDs (1), aspirin (1), statin (1), and use of PPI (2).

**TABLE 4:** Risk score for gastric cancer prediction.

Risk factor	Criteria	Points
Age in years		
	18–39	0
	40–49	2
	50–60	3
	61–70	4
Sex		
	Female	0
	Male	1
*Helicobacter pylori* infection		
	No	0
	Yes	1
Use of non‐steroidal anti‐inflammatory drugs		
	Yes	0
	No	1
Use of aspirin		
	Yes	0
	No	1
Use of statins		
	Yes	0
	No	1
Use of proton pump inhibitors		
	No	0
	Yes	2

The scoring system ranges from 0 to 11, and a subject's score was based on the sum of all the points allocated to each individual risk factor. A scoring of 0–8 was designated as “average risk (AR)”. Scores at 9 or above had prevalence remarkably higher than the overall prevalence, and hence were assigned as “high risk (HR)” (Table 5). From this stratification (Table 6), the prevalence of gastric cancer was 2.05% and 0.09%, respectively, for the HR and LR groups in the derivation cohort. Similarly, in the validation cohort, the prevalence of gastric cancer was 1.81% and 0.10%, respectively, for the HR and AR groups. The risk of gastric cancer in the HR group was significantly higher than that in the AR group (18.87, 95% CI 15.68–22.71, *p* < 0.001). The number needed to treat (NNT) was 58, and the number needed to refer (NNR) was 1041 for the AR group and 55 in the HR group.

**TABLE 5:** Distribution of number of subjects for each score category in the derivation cohort.

Score	Subjects, *n* (%)	Subjects with gastric cancer, *n* (%)	
0	0 (0.0)	0 (0.0)	Average risk
1	0 (0.0)	0 (0.0)	
2	27 (0.0)	0 (0.0)	
3	906,648 (20.9)	50 (0.0)	
4	788,331 (18.1)	40 (0.0)	
5	593,043 (13.6)	184 (0.0)	
6	901,376 (20.7)	733 (0.1)	
7	790,059 (18.2)	1528 (0.2)	
8	352,988 (8.1)	1564 (0.4)	
9	11,928 (0.3)	207 (1.7)	High risk
10	2797 (0.1)	95 (3.4)	
11	27 (0.0)	1 (3.7)	

**TABLE 6:** Prevalence of gastric cancer by risk tier.

Risk tier (risk score)	Derivation cohort		Validation cohort		
	Subjects, *n* (%)	Gastric cancer (%)	Subjects, *n* (%)	Gastric cancer (%)	Relative risk (95% CI)
		(95% CI)		(95% CI)	
Average risk (0–8)	4,332,472 (99.7)	4099 (0.09)	1,856,075 (99.7)	1783 (0.10)	1
		(0.09–0.10)		(0.09–0.10)	
High risk (9–11)	14,752 (0.3)	303 (2.05)	6398 (0.3)	116 (1.81)	18.87
		(1.83–2.28)		(1.49–2.14)	(15.68–22.71)
					*p* < 0.001
Total	4,347,224 (100)	4402 (0.10)	1,862,473 (100)	1899 (0.10)	
		(0.10–0.10)		(0.10–0.11)	

Abbreviation: CI, confidence interval.

### 3.5 Validity and reliability of the model

The Cochran–Armitage trend test showed that an increase in the proportion of gastric cancer as a function of the risk score was statistically significant. In addition, the Hosmer–Lemeshow goodness‐of‐fit statistics (*p* > 0.05) demonstrated the reliability of the final prediction algorithm, implying a close match between predicted risk and real risk.

## 4 DISCUSSION

### 4.1 Major findings and implications to clinical practice

In this study, we found that age, gender, previous history of *H. pylori* infection, the use of PPI, and the non‐use of NSAIDs, aspirin, and statin were independent predictors of gastric cancer in a large Chinese population. The risk algorithm has a high discriminatory capability and it could successfully predict gastric cancer. Although recent studies have identified that the use and non‐use of several medications as independent risk factors for gastric cancer, no clinical score that incorporates the chronic medication use for the prediction of gastric cancer has been developed. OGD appointments should be arranged for patients with high risks of gastric cancer. Nevertheless, current risk‐scoring algorithms have relatively modest discriminatory capabilities to stratify patients. Therefore, a more accurate risk prediction model of gastric cancer for patients is required to improve diagnostic efficiency and facilitate urgent referral of high‐risk subjects, particularly in regions with a scarcity of OGD resources. The developed algorithm provides physicians and patients with an estimation on gastric cancer risk, and informs shared decision making on the timing of OGD in clinical setting. This risk stratification strategy in our study could facilitate early detection of gastric cancer in high‐risk patients, increase the efficiency of screening, allow a better allocation of resource when planning OGD procedures, lead to a reduction in medical expenditure, and provide evidence for future guideline formulation on cost‐effective arrangement of OGD in patients suspected as having gastric cancer. Our findings may also result in a potential inclusion of a novel score in guidelines developed in the future.

### 4.2 Relationship with literature

A systematic review included 12 studies on risk prediction models for gastric cancer in the general population.^3^ The models have fair to good discriminatory capabilities with variables that can be easily obtained in clinical practice. However, less than half of the models were validated^3^ and the studies have a high risk of bias due to methodologic limitations. Furthermore, the studies had a very limited sample size as only one study has a large sample size of about 2.1 million patients.^17^ The number of predictors included in the studies ranged from 5 to 12 with a median of 7.5. Age^9^, ^17^, ^18^, ^19^, ^20^, ^21^, ^22^, ^23^ (used in 9 models (75%)) was the most frequently used risk predictors, followed by salt preference^9^, ^17^, ^18^, ^20^, ^22^ (*n* = 6, 50%), and *H. pylori* infection^9^, ^18^, ^21^, ^22^, ^23^, ^24^ (*n* = 6, 50%). Apart from these variables, all other predictors had a usage frequency of less than 50%. Although salt preference has been identified as a potential risk factor of gastric cancer,^25^ some studies used simple, subjective choices^17^ to categorize salt preference without a formal and recognized standard, or used the consumption of fish roe as a proxy for the measurement.^22^ These pose a challenge to evaluate the true effect of the predictors in the models. Some studies included common lifestyle habits such as smoking^17^, ^18^, ^22^, ^23^ (*n* = 5, 41.7%) and alcohol^17^, ^20^ (*n* = 3, 25%) in the prediction model. However, there has been a lack of consistent evidence supporting the associations between gastric cancer and tobacco smoking^26^ and alcohol drinking.^26^ It is also worthy to note that previous models with the highest prediction performance^19^, ^20^ were based predominately on lifestyle habits and demographic factors that were assessed only by questionnaires. Also, diagnostic indicators such as *H. pylori* infection were not included, despite results showing that approximately 89% of all gastric cancers could be attributable to *H. pylori* infection, indicating its potential in the estimation of gastric cancer risk.^27^

Despite the association between medications and gastric cancer, none of the studies included the use or non‐use of medication in the prediction models. In a meta‐analysis on NSAIDs, it is found that the risk of gastric cancer was 43% lower among regular users of NSAIDs than non‐users (OR = 0.57, 95% CI = 0.44–0.74).^6^ Users of aspirin experienced similar magnitude of risk reduction as research findings showed that regular users of aspirin for more than 3 years had significantly lower risk of gastric cancer (aHR = 0.40, 95% CI = 0.16–0.98).^7^ A nested case–control study found significantly lower odds of gastric cancer incidence for users of any statin, hydrophilic statins, or lipophilic statins (OR = 0.88, 95% CI = 0.81–0.86; OR = 0.78, 95% CI = 0.66–0.92; OR = 0.91, 95% CI = 0.84–0.99, respectively) after adjustment. On the contrary, the users of PPIs (>3 years) had more than 2 times (pooled OR = 2.45, 95% CI = 1.41–4.25)^28^ the risk of developing gastric cancer compared to non‐users. Inclusion of the chronic use of medication into a risk prediction model is not an uncommon practice, as previous studies have included the use of aspirin,^29^ NSAIDs^30^ and various other medications in the prediction model for other cancers.

In the current model, we selected age, gender, previous history of *H. pylori* infection, the use of PPI, and the non‐use of NSAIDs, aspirin, and statin in the scoring system. We excluded some chronic diagnoses (ischemic heart disease, cerebrovascular disease, and heart failure) due to their significant interaction effects with age. The predictors chosen to construct the model are objective, and this could minimize the effect of recall and information bias–allowing the risk prediction model practical and convenient for clinical use.

### 4.3 Strengths and limitations

This study has several strengths: (1) a large number of patients (more than 6 million) who have received upper digestive endoscopy were included, consisting of all patients in the general population, allowing a large validation cohort to evaluate the prediction accuracy and generalizability of the model; (2) a combination of potential risk factors were tested for the most accurate prediction of gastric cancer, while medication use were incorporated into the algorithm to enhance prediction accuracy. Nonetheless, there are a few limitations that should be addressed. First, a small proportion (around 1%) of the diagnostic codes were not available as they might have been included in the free text section of the electronic patient records, leading to difficult retrieval from CDARS. Second, the score did not include existing symptoms of gastric cancer into the model as they were unavailable in the disease coding system. Third, our findings may not be generalized to asymptomatic individuals who attend for screening as the dataset could only provide OGD results in symptomatic patients. Fourth, only the earliest OGD was used in our analysis to avoid over‐representation of a certain group of subjects. It is possible that GC cases in their later life are missed. Fifth, it is possible that due to the low testing rate of *H. pylori* in Hong Kong, the strength of the associations between other factors and gastric cancer may be impacted, cautions should be exercised when applying the algorithm to population with significant different prevalence of *H. pylori*. Finally, the use of a large sample size may have potential impact on the effectiveness of the statistical analysis. However, the inclusion of a substantial portion of the population allows for a more robust analysis and enhances the generalizability of the risk score.

## 5 CONCLUSION

In conclusion, this study has devised and validated an accurate and easy‐to‐use scoring algorithm with a high discriminatory capability to predict gastric cancer in symptomatic patients. Further studies can be conducted to examine its predictive performance among individuals in other populations.

## AUTHOR CONTRIBUTIONS

**Martin CS Wong:** Conceptualization (equal); project administration (equal); writing – original draft (equal); writing – review and editing (equal). **Eman Yee‐man Leung:** Data curation (equal); formal analysis (equal); writing – review and editing (equal). **Sarah TY Yau:** Data curation (equal); formal analysis (equal); writing – review and editing (equal). **Sze Chai Chan:** Writing – review and editing (equal). **Shaohua Xie:** Writing – review and editing (equal). **Wanghong Xu:** Writing – review and editing (equal). **Junjie Huang:** Writing – original draft (equal); writing – review and editing (equal).

## FUNDING INFORMATION

This research did not receive any specific grant from funding agencies in the public, commercial, or not‐for‐profit sectors.

## CONFLICT OF INTEREST STATEMENT

The authors declare no potential conflicts of interest.

## Supporting information


Figure S1.

## Data Availability

The data generated in this study are available upon request from the corresponding author.
