# Supplementary material for: Prediction algorithm for gastric cancer in a general population: A validation study
Source: Cancer Med. 2023 Oct 19;12(21):20544–53. doi: 10.1002/cam4.6629 (PMC10660462; doi:10.1002/cam4.6629)
Supplement: Supplementary file 1 — Figure S1. [file CAM4-12-20544-s001.docx]

**Scored and divided**

**into “Average Risk” & “High Risk” groups**

**6,209,697 Chinese patients with suspected gastric cancer**

**4,347,224 participants**

**as derivation cohort**

Pearson Chi-square test

**Variables with initial p<0.05 selected for binary logistic regression model**

Corresponding adjusted odds ratio (AOR) halved and rounded to the nearest integer

The sum of all the risk factors is the risk score

for each subject

Receiver operating

characteristic (ROC) curve & The area under the curve (AUC)

**Weighting assigned to each independent**

**variable in the risk score**

The score with a

magnitude ≤ overall

proportion of GC^1^

**Proportion of GC^1^**

**evaluated according**

**to each score**

Evaluate the predictive ability of the scoring system and discriminative

capability of the

prediction algorithm

Concordance (c)-statistics

**(AR)**

“**Average Risk**”

**1,862,473 participants as validation cohort**

The score with a magnitude > overall proportion of GC^1^

Evaluate the ability of the scoring system to predict the risk of having GC^1^

C-statistics and the area under the ROC curve^2^

**Assess the reliability of the final model**

**(p > 0.05 indicates good match)**

Hosmer-Lemeshow goodness-of-fit statistic

**(HR)**

**“High Risk”**

**Supplementary Figure 1 Development of a risk algorithm based on derivation and validation cohorts**

^1^GC: gastric cancer; ^2^ROC: Receiver Operating Characteristics

Random assignment (7:3) to

derivation and validation cohorts
